# Genetic changes during a laboratory adaptive evolution process that allowed fast growth in glucose to an *Escherichia coli* strain lacking the major glucose transport system

**DOI:** 10.1186/1471-2164-13-385

**Published:** 2012-08-10

**Authors:** César Aguilar, Adelfo Escalante, Noemí Flores, Ramón de Anda, Fernando Riveros-McKay, Guillermo Gosset, Enrique Morett, Francisco Bolívar

**Affiliations:** 1 Departamento de Ingeniería Celular y Biocatálisis, Instituto de Biotecnología. Universidad Nacional Autónoma de México (UNAM), Cuernavaca, Morelos, 62210, México; 2 Winter Genomics, México D.F. 07300, Cuernavaca, Morelos, 62210, México

**Keywords:** PTS system, Resequencing, Laboratory evolution, Mutation, Deletion, Adaptation, *rppH*, Glycolysis

## Abstract

**Background:**

*Escherichia coli* strains lacking the phosphoenolpyruvate: carbohydrate phosphotransferase system (PTS), which is the major bacterial component involved in glucose transport and its phosphorylation, accumulate high amounts of phosphoenolpyruvate that can be diverted to the synthesis of commercially relevant products. However, these strains grow slowly in glucose as sole carbon source due to its inefficient transport and metabolism. Strain PB12, with 400% increased growth rate, was isolated after a 120 hours adaptive laboratory evolution process for the selection of faster growing derivatives in glucose. Analysis of the genetic changes that occurred in the PB12 strain that lacks PTS will allow a better understanding of the basis of its growth adaptation and, therefore, in the design of improved metabolic engineering strategies for enhancing carbon diversion into the aromatic pathways.

**Results:**

Whole genome analyses using two different sequencing methodologies: the Roche NimbleGen Inc. comparative genome sequencing technique, and high throughput sequencing with Illumina Inc. GAIIx, allowed the identification of the genetic changes that occurred in the PB12 strain. Both methods detected 23 non-synonymous and 22 synonymous point mutations. Several non-synonymous mutations mapped in regulatory genes (*arcB*, *barA, rpoD, rna*) and in other putative regulatory loci (*yjjU*, *rssA* and *ypdA*). In addition, a chromosomal deletion of 10,328 bp was detected that removed 12 genes, among them, the *rppH*, *mutH* and *galR* genes. Characterization of some of these mutated and deleted genes with their functions and possible functions, are presented.

**Conclusions:**

The deletion of the contiguous *rppH*, *mutH* and *galR* genes that occurred simultaneously, is apparently the main reason for the faster growth of the evolved PB12 strain. In support of this interpretation is the fact that inactivation of the *rppH* gene in the parental PB11 strain substantially increased its growth rate, very likely by increasing glycolytic mRNA genes stability. Furthermore, *galR* inactivation allowed glucose transport by GalP into the cell. The deletion of *mutH* in an already stressed strain that lacks PTS is apparently responsible for the very high mutation rate observed.

## Background

Genome changes including point mutations, duplications, and recombination with homologous and heterologous DNA are the driving force of evolution. Bacteria, the most diverse and adapted types of cells in the biosphere, have permanently been evolving during millions of years to survive different environmental changes. Comparative genomics is a very powerful tool to analyze bacterial evolution occurring over short periods of time [1-3]. Moreover, whole genome resequencing of evolved *Escherichia coli* strains using simultaneously two different methodologies has recently been reported. This strategy is certainly very useful for understanding bacterial evolution, such as pathogen emergence, adaptation to environmental perturbations or during fermentation events used to generate derivative strains with enhanced industrial capacities [3-5].

**Figure 1:**
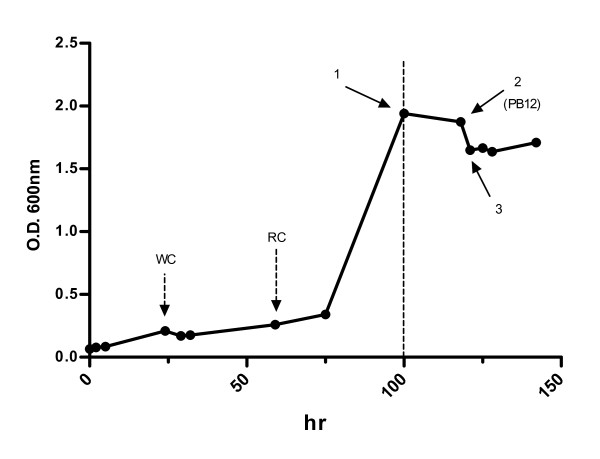
**Isolation of the evolved PB12 strain.** The isolation of PB12 has previously been reported and is included to provide orientation to the reader and for discussion purposes [10]. The evolutionary process that generated the PB12 strain initiated with the parental PB11 strain that lacks the PTS system. Deletion of this system generates a carbon stress response when PB11 is grown in glucose as the sole carbon source [9,13]. This strain that grows very slowly in glucose and generates white colonies (WC) in glucose-McConkey agar plates, was grown in a batch culture fermentor containing minimal medium with 2 g/l of glucose as the sole carbon source and 30 μg/ml of kanamycin. Under these conditions, a selection pressure is generated, favoring faster growing mutants. The culture was maintained until the stationary phase and then a continuous culture was initiated by feeding a glucose solution at progressively higher dilution rates in the same medium. Dotted line indicates the end of the batch culture and the start of the continuous culture. This procedure allowed the isolation of mutants according to their growth rates. Samples were monitored on glucose-McConkey agar plates to identify red colonies as an indicative of glucose utilization [Glc^+^ phenotype. Red colonies (RC) were detected after a period of 70 hr. The arrows indicate the isolation time for several Glc^+^ variants including PB12. Numbers indicate different dilution rates (D = h^-1^). All the isolated colonies from this culture carry the same large deletion present in strain PB12 (data not shown). This figure was derived and modified from figure 1 from Flores et al. 2007 [10]

We have constructed and characterized *Escherichia coli* strains that lack the phosphoenolpyruvate: carbohydrate phosphotransferase system (PTS), by deletion of the *ptsH*, *ptsI,* and *crr* genes, which is the major bacterial component involved in glucose transport and its phosphorylation. One of these strains, PB11, in spite of growing very slow in glucose (with a specific growth rate (μ) = 0.1 vs. 0.7 h^-1^ as compared to the parental strain JM101), accumulates high amounts of phosphoenolpyruvate, which can be diverted to the synthesis of aromatic compounds. PTS deletion results in a carbon stress response when the PB11 strain is grown in glucose as the sole carbon source that induces carbon scavenging. Strains lacking PTS can co-utilize several carbon sources due to the lack of catabolite repression exerted by PTS, and their glycolytic flux is reduced as part of a carbon limitation response [6-13]. As a metabolic engineering strategy, an adaptive laboratory evolution process for the selection of faster growing derivatives of the PB11 strain was carried out in a fermentor in minimal medium with glucose as the sole carbon source. In this process, after entering the stationary phase this carbohydrate was fed by progressively increasing the dilution rate. The resulting strain, PB12, which achieved a very reasonable growth rate (μ= 0.44 h^-1^), was selected in a process that lasted 120 hours (hr) (Figure 1) [9,10,12,13]. The evolved PB12 strain that in the absence of PTS uses the galactose permease (GalP), as the parental PB11 strain for glucose transport, has been utilized for overproduction of aromatic compounds [7,9,12,14-17]. 

It is well known that *E. coli* cells can adapt their metabolism to achieve higher growth rates as a result of specific mutations [2,5,18]. To get insights of the faster growth of the PB12 strain, we have compared its transcript levels with those of the parental PB11 strain, by reverse transcriptase quantitative real time PCR (RT-qPCR), of critical metabolic pathways. Interestingly, we found that all glycolytic and several other central carbon metabolism genes, including those that code for the tricarboxylic acid (TCA) cycle enzymes, are overexpressed, suggesting a very efficient carbon utilization by the evolved strain [7-13,19]. We have previously shown that a mutation in the *arcB* gene could be responsible for the overexpression of the TCA genes [9,20-22]. In addition a second mutation responsible of amber stop codon at position 98 in the *rpoS* gene which codes for the sigma factor RpoS, was detected in PB12 when compared against strain MG1655 [9,11]. Nevertheless, to get a detailed knowledge at the molecular level, of all the different genetic changes that occurred in the PB12 strain, a complete genomic analysis is required. This information will allow a better understanding of the basis of growth adaptation, plasticity, and the physiology of this evolved *E. coli* strain, and also will be useful in the design of improved laboratory adaptive evolution and metabolic engineering strategies for enhancing carbon diversion into the aromatic pathway utilizing strains lacking PTS.

In this work, using the Roche NimbleGen Inc. comparative genome sequencing technique (CGS) and high throughput sequencing with Illumina Inc. GAIIx, we identified all the genetic changes that occurred in the evolved PB12 strain during the selection process and analyzed and characterized the most relevant ones. Results of the whole genome sequencing, supported by transcript quantification by RT-qPCR and by knockout inactivation of selected genes in the parental PB11 strain, indicate that a simultaneous deletion of several contiguous genes including *rppH*, *mutH* and *galR*, is the main reason for the fast growth in glucose. *galR* codes for the repressor of the *gal* operon that includes *galP* that codes for the GalP permease [23], *rppH* codes for the RNA pyrophosphohydrolase (RppH), which initiates mRNA degradation [24], while *mutH* codes for the endonuclease of the MutHLS system involved in the mismatch DNA repair system [25]. In addition, several non-synonymous point mutations were detected as one located in the RNase I coding gene *rna*, involved in the degradation of RNA [26,27], while others were located in known and in putative regulatory genes, such as *arcB*[21,22]*, barA*[28,29]*rpoD*[30], *rssA*[31] and *yjjU*[32,33]. Finally, other mutation was mapped on *ypdA*, which code for a putative histidine kinase [34].

## Results and discussion

### Detection and characterization of non-synonymous point mutations in the evolved PB12 strain

Two comparative whole genome nucleotide sequence analyses of the evolved PB12, its parental JM101, and the wild type K-12 MG1655 strains were performed. The first was carried out by Roche NimbleGen Inc., Madison, WI (RN), using their CGS method; the second analysis was developed by Winter Genomics Inc., Mexico City (WG), using Illumina’s massively parallel sequencing technology (see Materials and Methods). In the RN analysis, 26 non-synonymous point mutations were detected in structural genes; 21 of them were also mapped at the same positions by WG. In addition, 6 non-synonymous mutations were detected only by WG (Table 1A and Tables S1 and S2 presented in Additional file 1 and Additional file 2). Since there was some discrepancies between the two technologies that utilized DNA obtained from the original frozen stock of the PB12 strain (see Materials and Methods), we decided to sequence each of the mutant genes solely reported by one company, after PCR amplification using the Sanger methodology. Only two of the mutants detected by WG (in the *csgF* and *ytfR* genes) could be confirmed by Sanger resequencing. The mutations in the other four genes (*ftsK, stfE, C0362* and *rsxC*) reported by WG and the five other genes (*mdlB*, *yagN*, *ydfN*, *ykfA* and *yagG*) reported by RN (Tables S1 and S2 presented in Additional file 1 and Additional file 2), could not be confirmed by the Sanger methodology (data not shown). Therefore, the total number of non-synonymous point mutations detected, comprised 21 reported by both companies, plus 2 additional mutations reported only by WG. 14 of the 21 common mutations including the ones located in regulatory genes (see below), were confirmed by Sanger resequencing (Table 1A). Recently, it has been shown that when both types of methodologies were utilized simultaneously for whole genome resequencing of *E. coli* strains in which growth adaptations by evolution occurred, both techniques reported false positive mutations [4,5]. Therefore, it is likely that the mutations in the PB12 strain not confirmed by the Sanger methodology are false positives. However, since the *mutH* gene deletion in this strain is responsible of increasing the mutation rate in *E. coli*[35] (see below), it could be possible that the two non-synonymous mutations detected only by WG, and confirmed by Sanger, are due to *de novo* changes that occurred in the overnight culture utilized to obtain DNA for genome analysis by WG and not in the fermentation process started with the PB11 strain (Figure 1). Alternatively, these two mutations could be real, but they were not detected by the RN analysis. Importantly, the nucleotide sequence of the parental PB11 strain was also determined by WG (data not shown). None of the point mutations that occurred in PB12 were detected in PB11, indicating that they appeared in the laboratory adaptive evolution process. 

**Table 1:** Mutations that occurred in the evolved PB12 strain during the adaptive process

**A)**	**Gene**	**Basic description**	**Mutations**			
		**Hypothetical genes**	**JM101 and PB11**	**PB12**	**Nucleotide**	**Pos/Change**
	gfcD*	Hypothetical lipoprotein.	GTA	cTA	916	306 V-L
	yafV*	Predicted C-N hydrolase family amidase, NAD(P)-binding.	CGC	CaC	452	151 R-H
	+*ytfR*^(WG)^	Putative ATP-binding component of a galactose ABC transporter.	GTC	GcC	602	201 V-A
		**Metabolism and Transport**	**JM101 and PB11**	**PB12**	**Nucleotide**	**Pos/Change**
	+actP*	Acetate/glycolate permease in the solute:sodium symporter (SSS) family.	GTA	GcA	731	244 V-A
	arnT*	L-Ara4N transferase catalyzes addition of L-Ara4N to lipid A under some conditions.	TAC	TgC	1193	398 Y-C
	+chbC*	Integral membrane transport protein, member of the chitobiose PTS transporter.	GCG	GtG	347	116 A-V
	+*csgF*^(WG)^	Curli secretion and assembly complex.	AAU	AgU	89	30 N-S
	+dgoT*	Probable galactonate transporter, member of the major facilitator superfamily (MFS) of transporters.	GAT	GgT	653	218 D-G
	dhaM*	Dihydroxyacetone kinase subunit M, homologous to certain PTS components.	TGG	TGa	1038	346 W-stop
	+dppF*	ATP-binding component of the dipeptide ABC transporter.	CAC	CgC	680	227 H-R
	fdhD*	Protein with unknown function, required for wild-type formate dehydrogenase-N activity.	AGT	gGT	652	218 S-G
	fimH*	Minor fimbrial subunit, D-mannose specific adhesin, subunit of fimbrial complex.	ACT	gCT	535	179 T-A
	glpT*	Major uptake transporter for glycerol-3-phosphate, belongs to the major facilitator superfamily (MFS).	CCG	CtG	416	139 P-L
	rfe*	Undecaprenyl-phosphate α-N-acetylglucosaminyl transferase .	GCC	aCC	238	80 A-T
	+sucA*	E1(0) component of the oxoglutarate dehydrogenase complex.	GAT	GAa	372	124 D-E
	ydiQ*	Putative subunit of YdiQ-YdiR flavoprotein.	GTG	GcG	218	73 V-A
		**Regulatory and putative regulatory genes**	**JM101 and PB11**	**PB12**	**Nucleotide**	**Pos/Change**
	+arcB*	**Hybrid protein sensory kinase, member of two-component system.**	**TAC**	**TgC**	**212**	**71 Y-C**
	+barA*	**Hybrid protein sensory kinase, member of two-component system.**	**TTC**	**cTC**	**1096**	**366 F-L**
	+rna*	**RNase I, cleaves phosphodiester bond between any two nucleotides.**	**GCC**	**aCC**	**268**	**90 A-T**
	+rpoD*	Sigma 70 factor, subunit of RNA polymerase.	GTT	aTT	1744	582 V-I
	+rssA*	**Hypothetical protein, coded by a gene that is part of the*rssAB*operon.**	**CGC**	**CaC**	**773**	**258 R-H**
	+yjjU*	**Conserved hypothetical protein, could work as a transcriptional protein.**	**ACT**	**gCT**	**338**	**179 T-A**
	+ypdA*	**Predicted sensory kinase member of two-component system.**	**GCG**	**tCC**	**598**	**200 A-S**
**B)**	**Gene**	**Basic description**	**Mutations**			
	*ptsP*	Member of a second PTS chain involved in nitrogen metabolism	Present	Absent	---	---
	***rppH***	**RNA pyrophosphohydrolase that initiates mRNA degradation by hydrolysis of the 5'-triphosphate end.**	**Present**	**Absent**	**---**	**---**
	*ygdT*	Hypothetical protein.	Present	Absent	---	---
	***mutH***	**dGATC endonuclease in the MutHLS complex, the methyl-directed mismatch repair pathway.**	**Present**	**Absent**	**---**	**---**
	*ygdQ*	Putative transport protein.	Present	Absent	---	---
	*ygdR*	Predicted protein.	Present	Absent	---	---
	*tas*	Putative NAD(P)-linked reductase that acts in starvation-associated mutations.	Present	Absent	---	---
	*lplT*	Lysophospholipid transporter (LplT).	Present	Absent	---	---
	*aas*	2-acylglycerophosphoethanolamine acyltransferase/acyl-ACP synthetase.	Present	Absent	---	---
	*omrA*	Small RNA that is involved in regulating the protein composition of the outer membrane.	Present	Absent	---	---
	*omrB*	Small RNA that is involved in regulating the protein composition of the outer membrane.	Present	Absent	---	---
	***galR***	**DNA-binding transcription factor; represses transcription of the operons involved in transport and catabolism of D-galactose.**	**Present**	**Absent**	**---**	**---**

**Figure 2:**
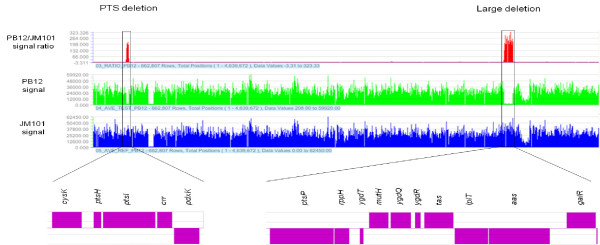
**Comparative genomic maps of the JM101 and PB12 strains.** Deletions detected in the PB12 strain. The small deletion is the result of the elimination of the PTS genes (*ptsH*, *ptsI* and *crr*) that was previously generated in the parental PB11 strain [7,9]. The largest of these deletions appeared during the laboratory adaptive evolution process (Figures 3 and 4).

**Figure 3:**
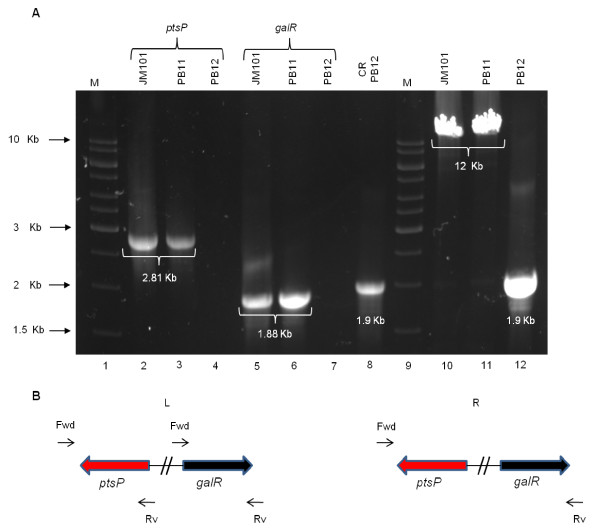
**Chromosomal deletion markers in the PB12 strain.** The absence of a chromosomal fragment in the PB12 strain was confirmed by PCR and by Sanger resequencing. Ten genes were deleted and the *galR* and *ptsP* genes were fused. Section **A** shows the *ptsP* and *galR* genes that were amplified in the JM101, PB11 and PB12 strains: line 1, (M) molecular weight markers; lines 2, 3 and 4, *ptsP* amplification in the JM101, PB11 and PB12 strains, respectively; lines 5, 6 and 7, *galR* amplification in the JM101, PB11 and PB12 strains, respectively; line 8, amplification of the chromosomal region in the PB12 strain; line 9, (M) molecular weight markers; lines 10, 11 and 12, amplification of the chromosomal region using DNA from strains JM101, PB11 and PB12, respectively. Section **B** presents the oligonucleotides utilized for DNA amplifications. The left section (L) includes the oligonucleotides employed for the amplification of the *ptsP* and *galR* genes of the three strains (lines 2–7), and the right section (R) presents the entire chromosomal regions of the same three strains amplified using ptsP-fwd and galR-rv oligonucleotides (lines 8, 10–12). The nucleotide sequences of the oligos utilized are included in table S3 presented in additional file 4.

**Figure 4:**
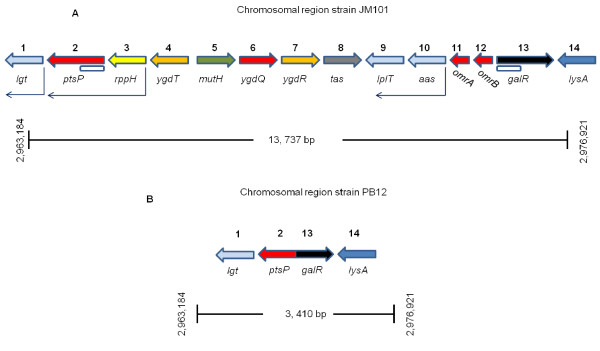
**Chromosomal gene arrangement in the parental JM101 strain and in the evolved PB12 derivative. A**) Gene organization in the chromosome of the parental strain. **B**) Gene deletion and chromosome rearrangement in the PB12 strain genome. Figure S1 presented in Additional file 3, includes the nucleotide sequence of the genomic region where the deletion occurred.

Table 1A presents the 23 non synonymous point mutations in structural genes that changed the code for a different amino acid when compared to the parental PB11 and JM101 strains genomes. The asterisks in the table indicate the 21 non-synonymous point mutations detected by both Roche NimbleGen Inc. (RN) and Winter Genomics Inc. (WG) methods. In addition 22 synonymous point mutations in different genes were also detected (Tables S1 and S2 in Additional file 1 and Additional file 2). The regulatory and possible regulatory genes analyzed in this study are in bold letters. The mutations in these seven regulatory and possible regulatory genes and in seven additional genes were confirmed by the Sanger methodology and are labeled with a +. 26 (21+5) point mutations in 26 genes were detected by RN, 5 of them were false positive. 27 (21+6) point mutations in 27 genes were detected by WG. In addition to the 21 mutations shared with RN, only two of these point mutations (noted as WG) were confirmed by the Sanger methodology. Additional file 1 and Additional file 2 (Tables S1 and S2) include the data from RN and WG. Table 1B presents the absent genes in the largest deletion reported by both companies. (Figures. 2, 3, 4 and S1, presented in Additional file 3). Basic descriptions of these genes were taken from www.ecocyc.org.

Among the non-synonymous substitutions detected some were located at genes with regulatory functions: *arcB*, *barA*, *rpoD*, *rna,* and three in putative regulatory genes: *yjjU*, *rssA* and *ypdA* (Table 1A). The mutation in the *arcB* gene, a tyrosine to cysteine residue substitution at position 71 that apparently modifies the ArcA/B ability to function as a repressor, has previously been reported by our group. We have proposed that this modification could be responsible for the overexpression of the TCA genes in the PB12 derivative as compared to the parental PB11 strain [9,20,21] (Table 1A). A new role for this mutation is proposed (see next sections).

The *barA* mutation resulted in a phenylalanine to leucine residue substitution at position 366 (Table 1A). This residue is located between two functional subregions of the HK domain of this protein; the “H box”, where the conserved histidine residue involved in the autophosphorylation of BarA is located, and the “N box”, involved in ATP binding [28,29].

The change in the *rpoD* gene resulted in a valine to isoleucine residue substitution at position 582, which is located in a helix-turn-helix (HTH) motif of the RpoD coded protein (Table 1A). This motif is involved in the binding to the −35 promoter region, but it is unlikely that the conservative nature of the substitution, and the fact that this residue does not make any direct contact with the DNA [36], had any significant effect on promoter recognition.

The mutation in the *rna* gene, an alanine to threonine residue substitution at position 90 of the coded RNase I, is also unlikely to have any consequence, since it is located in a nonconserved structural part outside the catalytic region [37].

The *yjjU* mutation resulted in a threonine to alanine residue substitution at position 179 in the coded YjjU protein (Table 1A). It has been proposed that this protein could have a regulatory role [32,33].

The mutation in the *rssA* gene resulted in an arginine to histidine residue substitution at position 258 of its product. The importance of this mutation, as well as the function of the RssA protein, are unknown; however, it might be functionally related to the RssB protein, which is involved in the degradation of the RpoS, since both conserved proteins are coded by genes located in the same operon [31]. An esterase function has also been predicted for RssA [Uniprot.org].

The point mutation in the *ypdA* gene located at position 200, caused an alanine to serine residue substitution. This gene codes for a predicted sensory histidine kinase of the two-component system YpdA-YpdB [34].

The products of most of the other mutated genes (*sucA*, *dhaM*, *ydiQ*, *chbC*, *glpT*, *arnT*, *dppF*, *rfe*, *fdhD*, *fimH*, *csgF*, *dgoT,* and *actP*) detected by both companies and the two mutations detected only by WG (*csgF* and *ytfR*), are involved in bacterial metabolism and transport (Table 1A).

In addition, 22 synonymous mutations were reported; 16 of them detected by both companies. These mutations were not analyzed since it is unlikely that they could have any significant effect on the phenotype (Tables S1 and S2 in Additional file 1 and Additional file 2). Also, several point mutations were detected in non-coding regions by WG. It should be noted that these unconfirmed mutations are unlikely to be located in regulatory regions (data not shown).

The presence of a mutation in the *rpoS* gene in the PB12 strain has been reported. This change generates a stop codon instead of a glutamine coding residue at position 98. It is known, that this strain being a derivative of JM101, carries a *supE* mutation, which suppresses amber stop codons [11,38,39]. Originally this mutation was considered to have occurred during the adaptive evolution process since the change was detected when compared against the sequence of strain MG1655 [9,11,40]. However, both comparative genome sequencing strategies showed that this mutation was already present in the parental PB11 and the JM101 strains. Sanger resequencing confirmed the presence of this mutation in both of the parental strains (data not shown). So, this mutation was incorporated at sometime during the development of the JM101 strain from its parental strains JC3130, CSH51 and 71.18 [38,39].

### Detection, characterization of a chromosomal deletion in the evolved PB12 strain, and analysis of the effects of the deleted genes

A deletion of 10,328 bp located at minute 64 on the chromosome of the PB12 strain that removed simultaneously the *rppH*, *ygdT*, *mutH*, *ygdQ*, *ygdR*, *tas*, *lplT*, *aas*, *omrA*, *omrB,* and the part of *ptsP* and *galR* genes, was detected by both RN and WG analyses (Table 1B and Figure 2). This deletion was confirmed by PCR (Figure 3) and its limits mapped within the *ptsP* and *galR* genes, resulting in a fusion of the remaining segments of these two genes (Figure 4). The nucleotide sequence of the fused fragments was confirmed by Sanger and it is presented in Additional file 3 (Figure S1). Neither repeated sequences nor insertion sequences were detected in the chromosomal DNA regions flanking the deleted genes; therefore, the molecular bases of this deletion are unknown.

The analyses of three of these genes, *galR*, *mutH* and *rppH* whose deletion could be the main cause (see below) of the faster growth in glucose of the evolved PB12 strain is presented and discussed in the following sections.

The *galR* gene codes for the repressor of the *gal* regulon that includes the *galP* gene [23]. Therefore, the inactivation of this gene in the PB12 strain is apparently responsible for the high transcription levels of most of the *gal* genes. In agreement with this proposition, we have previously demonstrated that the PB12 strain that lacks PTS is dependent on GalP for glucose transport [9,12].

The *rppH* gene codes for an RNA pyrophosphohydrolase that initiates mRNA degradation by hydrolysis of the 5’ triphosphate-end. After this modification, RNase E can initiate further mRNA degradation. It has been reported that the level of 382 transcripts increased significantly in *E. coli* cells lacking RppH [24]. Accordingly, in the PB12 strain higher transcript levels of many genes, among them glycolytic and TCA genes were detected by RT-qPCR as compared to the parental JM101 and PB11 strains. We could not explain the basis of the simultaneous “overexpression” of the genes involved in these metabolic pathways (with the exception of the TCA cycle genes due to the *arcB* mutation, as previously mentioned) [9,11]. In the light of the phenotype of the strains lacking *rppH*, it is likely that the higher levels of the transcripts observed in the PB12 strain is a result of impaired mRNA degradation instead of overexpression (see below). In fact, *rppH* inactivation in the PB11Δ*rppH* strain increased its μ 261% (Table 2). In agreement with this μ increment, as it will be presented in detail in the next sections, the RT-qPCR values of most central metabolic genes were increased 1.5- to 18-fold when the *rppH* gene was inactivated in the PB11Δ*rppH* derivative as compared to the parental PB11 strain, allowing an improved glucose metabolism, probably due to higher levels of central metabolic genes transcripts (see next sections). Interestingly, in contrast to the PB11 strain, inactivation of the *rppH* gene in the parental JM101Δ*rppH* strain decreased 27% its μ (data not shown), indicating that this mutation disrupted the glucose metabolism in this strain that does not have impaired the glucose transport. From these results it is tempting to speculate that inactivation of *rppH* could be considered as a tool for increasing the half-life of certain mRNAs families, and therefore growth rates, in strains with certain limited growth conditions. 

**Table 2:** Specific growth rates (μ) of the PB11 strain and its derivatives

**Regulatory genes**		
**Strain**	**μ (h**^**-1**^**)**	**%**
PB11	0.13 ± 0.001	100
PB11Δ*barA*	0.12 ± 0.001	92
PB11Δ*rppH*	0.34 ± 0.019	261
PB11ΔrpoS*	0.06	46
PB11ΔarcA*	0.32	246
**Possible regulatory genes**		
**Strain**	**μ (h**^**-1**^**)**	**%**
PB11Δ*yjjU*	0.16 ± 0.005	123
PB11Δ*rssA*	0.17 ± 0.003	130
PB11Δ*rna*	0.13 ± 0.007	100
PB11Δ*ypdA*	0.13 ± 0.002	100

*These values have been previously reported [9-11].

The absence of the *mutH* gene, which codes for the MutH endonuclease that is part of the MutHLS complex involved in the DNA mismatch repair pathway, is probably responsible for the appearance of the large number of mutations detected in the PB12 strain during the short term adaptive laboratory evolution process that lasted for only 120 hr [5,7,9,10,12,18,25]. It is known that the absence of the *mutH* gene increases the mutation frequency in *E. coli* at least 200 fold [35]. In addition, starvation-induced mutagenesis among hundreds of *E. coli* natural isolates is increased on average 7-fold, but in certain strains up to 1000-fold [41-43]. The presented and analyzed information indicates that the 21 shared non-synonymous point mutations as well as the deletion are real mutations that appeared after a 120 hr period in this adaptive laboratory evolution process. A possible explanation for this high number of mutations is that the deletion of the *mutH*, *rppH* and *galR* genes occurred early in the very short laboratory evolutionary process allowing faster growth by itself. In the absence of *mutH*, some or several of the point mutations could then have occurred during a few replication cycles, favouring the selection of even faster growing variants in glucose. In addition, in this genetic background in which the PTS deletion generates a carbon stress response, further mutagenesis induction is expected [9,10,12,41,42,44,45].

### RT-qPCR mRNA expression values of relevant mutated genes and of central metabolic genes in the evolved PB12 strain

Table 3 shows mRNA expression values of relevant (mainly regulators or possible regulators) mutated genes and of some other genes as controls determined by RT-qPCR (see Materials and Methods). RT-qPCR values of more than 100 genes from the evolved PB12 strain, the parental PB11 and JM101 strains have previously been reported by our group, including the *arcB* gene in which a non-synonymous point mutation appeared, and are presented in Table 3 for comparison and analyses purposes [9-11]. Results indicate that the RT-qPCR values of some of the regulatory and possible regulatory genes were not substantially modified (except for *rpoS* and *barA*) with respect to the JM101 parental strain. As anticipated, except for *galR,* no transcripts of the 12 deleted contiguous genes were detected. 

**Table 3:** RT-qPCR values of central metabolism and regulatory genes

**Gene**	PB11*	PB12*	**JM101**Δ**rppH**	**PB11**Δ**rppH**	**PB11**ΔrpoS*	**PB12**ΔrpoS*	**PB11**ΔarcA*
	**Glycolysis**						
*aceE +*	0.40 ± 0.02	1.40 ± 0.02	1.57 ± 0.23	1.32 ± 0.21	0.20 ± 0.00	0.85 ± 0.00	0.40 ± 0.00
*aceF +*	0.60 ± 0.00	1.20 ± 0.00	2.66 ± 0.51	1.00 ± 0.12	0.40 ± 0.10	0.70 ± 0.00	0.60 ± 0.00
*eno*	0.50 ± 0.10	0.50 ± 0.14	0.89 ± 0.01	1.28 ± 0.41	0.20 ± 0.00	0.37 ± 0.00	0.30 ± 0.00
*fbaA*	0.90 ± 0.00	1.10 ± 0.05	0.82 ± 0.08	1.46 ± 0.18	0.90 ± 0.00	0.37 ± 0.10	0.40 ± 0.00
*gapA*	0.40 ± 0.10	1.70 ± 0.20	6.98 ± 1.17	3.76 ± 0.67	0.10 ± 0.00	1.40 ± 0.20	0.30 ± 0.00
*glk*	1.00 ± 0.00	2.20 ± 0.10	3.43 ± 0.72	6.04 ± 0.03	0.70 ± 0.10	1.70 ± 0.10	0.80 ± 0.10
*gpmA*	0.90 ± 0.00	1.80 ± 0.00	3.07 ± 0.21	2.46 ± 0.13	0.60 ± 0.10	1.70 ± 0.30	0.50 ± 0.00
*lpdA +*	1.10 ± 0.10	2.00 ± 0.10	2.33 ± 0.00	3.16 ± 0.36	1.30 ± 0.30	1.70 ± 0.10	2.00 ± 0.10
*pfkA*	0.30 ± 0.00	0.50 ± 0.00	1.49 + 0.11	1.48 + 0.11	0.00 ± 0.00	0.20 ± 0.00	0.10 ± 0.00
*pgi*	1.00 ± 0.10	6.60 ± 0.30	3.47 ± 0.10	7.71 ± 0.91	0.40 ± 0.10	3.70 ± 0.10	0.40 ± 0.00
*pgk*	0.70 ± 0.00	1.20 ± 0.00	1.24 ± 0.20	1.08 ± 0.11	N.D.	N.D.	0.80 ± 0.00
*ppc*	0.60 ± 0.05	0.80 ± 0.00	3.81 ± 0.27	1.97 ± 0.40	0.80 ± 0.10	0.50 ± 0.10	N.D.
*pykA*	0.40 ± 0.00	1.00 ± 0.00	1.16 ± 0.12	0.83 ± 0.03	0.10 ± 0.00	0.51 ± 0.10	0.50 ± 0.10
*pykF*	0.80 ± 0.00	0.90 ± 0.00	0.89 ± 0.31	2.00 ± 0.26	0.20 ± 0.00	0.50 ± 0.10	0.40 ± 0.00
*tpiA*	0.50 ± 0.00	1.80 ± 0.00	4.84 ± 0.12	8.83 ± 2.61	0.20 ± 0.00	0.30 ± 0.00	0.40 ± 0.00
**Gene**	**TCA cycle**						
*acnA*	5.20 ± 0.60	5.70 ± 0.20	2.88 ± 0.31	10.80 ± 1.63	0.70 ± 0.10	3.50 ± 0.30	4.30 ± 0.20
*acnB +*	1.70 ± 0.36	1.70 ± 0.42	1.76 ± 0.06	24.80 ± 2.18	0.20 ± 0.00	0.70 ± 0.00	2.60 ± 0.30
*fumA +*	3.30 ± 0.67	3.60 ± 0.67	2.62 ± 0.72	24.07 ± 3.41	2.30 ± 0.50	3.80 ± 0.10	2.10 ± 0.10
*gltA +*	0.80 ± 0.10	1.30 ± 0.20	1.73 ± 0.31	6.68 ± 0.13	1.30 ± 0.20	1.20 ± 0.20	1.70 ± 0.10
*icdA +*	1.00 ± 0.10	1.90 ± 0.00	1.03 ± 0.11	4.66 ± 0.78	1.20 ± 0.00	2.40 ± 0.00	1.30 ± 0.00
*mdh*	1.20 ± 0.10	1.80 ± 0.50	1.93 ± 0.16	3.64 ± 0.32	1.40 ± 0.30	1.40 ± 0.10	1.10 ± 0.10
*sdhA +*	0.90 ± 0.00	2.00 ± 0.30	4.75 ± 0.98	5.96 ± 0.67	3.70 ± 0.20	4.00 ± 0.00	2.60 ± 0.10
*sdhB +*	0.80 ± 0.00	1.70 ± 0.00	4.40 ± 0.86	8.05 ± 0.86	2.20 ± 0.00	2.40 ± 0.30	1.30 ± 0.00
*sucA +*	1.40 ± 0.10	1.70 ± 0.10	3.28 ± 0.20	5.42 ± 1.39	1.20 ± 0.20	1.00 ± 0.00	1.20 ± 0.00
*sucB +*	0.80 ± 0.00	1.40 ± 0.10	3.31 ± 0.62	8.28 ± 1.91	N.D.	N.D.	1.20 ± 0.00
*sucC +*	0.90 ± 0.10	1.60 ± 0.00	1.99 ± 0.43	7.41 ± 1.21	1.30 ± 0.10	1.50 ± 0.00	1.20 ± 0.20
*sucD +*	1.00 ± 0.20	1.40 ± 0.30	1.89 ± 0.32	7.16 ± 0.11	1.70 ± 0.20	1.00 ± 0.10	1.10 ± 0.10
**Gene**	**Gluconeogenic, glyoxylate and*poxB*shunt**						
*aceA +*	12.00 ± 1.20	1.90 ± 0.20	1.66 ± 0.38	24.20 ± 3.16	9.10 ± 2.80	0.80 ± 0.10	8.81 ± 1.00
*aceB +*	15.60 ± 2.50	3.70 ± 0.30	2.54 ± 0.09	28.65 ± 5.91	11.00 ± 2.10	0.74 ± 0.00	24.20 ± 2.60
*acs*	5.60 ± 0.74	8.00 ± 0.13	1.83 ± 0.19	21.82 ± 3.20	4.10 ± 0.10	3.40 ± 0.10	11.90 ± 0.10
*glcB +*	11.70 ± 1.4	3.70 ± 0.50	1.04 ± 0.28	12.62 ± 0.93	2.50 ± 0.20	1.80 ± 0.20	7.60 ± 0.30
*maeB*	1.20 ± 0.20	2.50 ± 0.03	1.18 ± 0.26	10.06 ± 1.19	1.60 ± 0.00	1.70 ± 0.00	N.D.
*pckA*	0.80 ± 0.06	2.30 ± 0.02	0.83 ± 0.21	2.81 ± 0.89	0.90 ± 0.20	1.30 ± 0.10	N.D.
*poxB*	4.20 ± 0.35	5.70 ± 0.48	3.30 ± 0.48	5.30 ± 0.56	1.50 ± 0.10	0.50 ± 0.00	2.80 ± 0.90
*ppsA*	3.70 ± 0.21	2.40 ± 0.09	7.34 ± 2.08	5.57 ± 1.34	3.30 ± 0.20	1.10 ± 0.20	N.D.
*sfcA*	1.90 ± 0.33	1.70 ± 0.10	2.56 ± 0.85	3.72 ± 0.28	1.10 ± 0.10	1.00 ± 0.00	N.D.
**Gene**	***gal*regulon**						
*galE*	38.80 ± 6.80	46.60 ± 6.50	0.53 ± 0.12	40.25 ± 1.97	1.40 ± 0.10	25.00 ± 2.10	N.D.
*galK*	39.00 ± 2.30	48.21 ± 4.00	2.12 ± 0.09	43.78 ± 3.43	N.D.	N.D.	N.D.
*galM*	8.10 ± 3.94	3.30 ± 0.70	0.96 ± 0.18	2.67 ± 0.85	N.D.	N.D.	N.D.
*galP*	12.40 ± 1.08	13.10 ± 1.86	1.98 ± 0.36	25.11 ± 0.98	N.D.	N.D.	11.70 ± 1.90
galR***	3.20 ± 0.10	1.20 ± 0.40	2.04 ± 0.25	5.20 ± 0.10	N.D.	N.D.	N.D.
*galS*	4.90 ± 0.47	3.20 ± 0.52	4.14 ± 0.35	23.48 ± 0.07	N.D.	N.D.	N.D.
*galT*	35.60 ± 2.37	42.50 ± 4.00	1.10 ± 0.12	64.34 ± 5.20	N.D.	N.D.	N.D.
*galU*	1.70 ± 0.30	2.10 ± 0.36	1.27 ± 0.01	2.36 ± 0.22	N.D.	N.D.	N.D.
**Gene**	**Respiration**						
*cyoA +*	0.60 ± 0.01	1.40 ± 0.07	2.16 ± 0.07	4.33 ± 0.38	N.D.	N.D.	1.10 ± 0.10
*cyoB +*	0.30 ± 0.00	1.40 ± 0.10	2.42 ± 0.58	3.11 ± 0.04	N.D.	N.D.	N.D.
*cyoC +*	0.30 ± 0.00	1.20 ± 0.20	2.93 ± 0.19	2.77 ± 0.55	N.D.	N.D.	N.D.
*cyoD +*	0.30 ± 0.02	1.10 ± 0.10	1.90 ± 0.43	3.05 ± 0.03	N.D.	N.D.	N.D.
*nuoA +*	0.40 ± 0.00	2.40 ± 0.10	3.53 ± 0.54	9.67 ± 1.98	N.D.	N.D.	1.00 ± 0.00
*nuoF +*	0.40 ± 0.10	1.40 ± 0.10	4.68 ± 0.11	6.84 ± 0.28	N.D.	N.D.	N.D.
*nuoN +*	0.40 ± 0.02	1.00 ± 0.08	0.44 ± 0.02	1.55 ± 0.18	N.D.	N.D.	N.D.
*ubiE +*	0.40 ± 0.00	1.60 ± 0.00	4.39 ± 1.31	12.01 ± 1.26	N.D.	N.D.	N.D.
**Gene**	**ppGpp metabolism**						
*gpp*	1.40 ± 1.20	5.20 ± 0.30	9.17 ± 1.65	4.77 ± 0.93	0.00 ± 0.00	0.10 ± 0.00	N.D.
*ndk*	0.40 ± 0.00	3.10 ± 0.10	3.49 ± 0.52	11.08 ± 0.43	0.50 ± 0.00	3.30 ± 0.10	N.D.
*ppa*	1.00 ± 0.00	6.10 ± 1.40	7.42 ± 0.33	9.29 ± 0.32	0.50 ± 0.10	3.80 ± 0.10	N.D.
*relA*	1.30 ± 0.00	1.60 ± 0.20	3.13 ± 0.20	1.51 ± 0.00	0.90 ± 0.10	1.10 ± 0.00	N.D.
*spoT*	1.10 ± 0.20	4.80 ± 0.20	6.00 ± 0.77	7.36 ± 0.82	0.50 ± 0.10	0.70 ± 0.10	N.D.
**Gene**	***pts*operon**						
*crr*	0.00 ± 0.00	0.00 ± 0.00	2.46 ± 0.37	0.00 ± 0.00	0.00 ± 0.00	0.00 ± 0.00	0.00 ± 0.00
*ptsH*	0.00 ± 0.00	0.00 ± 0.00	1.07 ± 0.12	0.00 ± 0.00	0.00 ± 0.00	0.00 ± 0.00	0.00 ± 0.00
*ptsI*	0.00 ± 0.00	0.00 ± 0.00	1.34 ± 0.14	0.00 ± 0.00	0.00 ± 0.00	0.00 ± 0.00	0.00 ± 0.00
**Gene**	**Genes in deleted chromosomal fragment in PB12**						
galR***	3.20 ± 0.10	1.20 ± 0.40	2.04 ± 0.25	5.20 ± 0.10	N.D.	N.D.	N.D.
lplT***	3.95 ± 0.53	0.00 ± 0.00	0.95 ± 0.12	2.83 ± 0.32	N.D.	N.D.	N.D.
mutH***	2.76 ± 0.02	0.00 ± 0.00	16.49 ± 0.22	30.48 ± 0.00	N.D.	N.D.	N.D.
rppH***	1.84 ± 0.20	0.00 ± 0.00	0.00 ± 0.00	0.00 ± 0.00	N.D.	N.D.	N.D.
ptsP***	1.80 ± 0.20	0.00 ± 0.00	0.55 ± 0.07	0.30 ± 0.06	N.D.	N.D.	N.D.
tas***	1.31 ± 0.07	0.00 ± 0.00	1.07 ± 0.21	12.80 ± 2.44	N.D.	N.D.	N.D.
ygdQ***	8.86 ± 1.86	0.00 ± 0.00	1.92 ± 0.06	6.10 ± 1.58	N.D.	N.D.	N.D.
**Gene**	**Genes with point mutations in PB12**						
arcB**	2.30 ± 0.70	1.30 ± 0.72	0.99 ± 0.06	1.23 ± 0.19	N.D.	N.D.	N.D.
barA**	4.14 ± 0.91	3.46 ± 0.46	3.25 ± 0.09	2.30 ± 0.53	N.D.	N.D.	N.D.
rna**	0.89 ± 0.18	3.40 ± 0.75	2.06 + 0.42	8.03 ± 0.00	N.D.	N.D.	N.D.
rssA**	4.89 ± 1.27	4.04 ± 0.43	6.45 ± 0.31	6.07 ± 0.95	N.D.	N.D.	N.D.
rpoD**	1.00 ± 0.20	0.90 ± 0.20	1.00 ± 0.20	1.18 ± 0.24	0.70 ± 0.20	1.10 ± 0.10	N.D.
*rpoS +*	2.90 ± 0.86	2.20 ± 0.20	2.03 ± 0.07	4.62 ± 0.34	0.10 ± 0.00	0.20 ± 0.00	2.60 ± 0.10
yjjU**	0.86 ± 0.26	8.51 ± 0.00	1.44 ± 0.03	4.31 ± 0.35	N.D.	N.D.	N.D.
ypdA**	1.59 ± 0.49	1.38 ± 0.15	1.20 ± 0.31	1.36 ± 0.11	N.D.	N.D.	N.D.
**Gene**	**Regulators**						
*arcA*	1.02 ± 0.02	1.30 ± 0.09	1.68 ± 0.49	3.86 ± 0.60	0.70 ± 0.00	1.10 ± 0.10	0.00 ± 0.00
arcB**	2.30 ± 0.70	1.30 ± 0.72	0.99 ± 0.06	1.23 ± 0.19	N.D.	N.D.	N.D.
barA**	4.14 ± 0.91	3.46 ± 0.46	3.25 ± 0.09	2.30 ± 0.53	N.D.	N.D.	N.D.
*crp*	0.80 ± 0.02	1.00 ± 0.05	1.38 ± 0.01	2.22 ± 0.64	N.D.	N.D.	N.D.
*cyaA*	1.40 ± 0.10	1.80 ± 0.10	2.37 ± 0.78	3.62 ± 0.68	N.D.	N.D.	N.D.
*fadR*	0.60 ± 0.03	1.60 ± 0.08	3.52 ± 0.03	3.34 ± 0.19	N.D.	N.D.	N.D.
*fruR*	1.30 ± 0.00	1.50 ± 0.07	1.41 + 0.02	4.34 + 0.34	N.D.	N.D.	N.D.
galR***	3.20 ± 0.10	1.20 ± 0.40	2.04 ± 0.25	5.20 ± 0.10	N.D.	N.D.	N.D.
*iclR*	0.40 ± 0.03	1.40 ± 0.07	0.81 ± 0.20	2.62 ± 0.39	N.D.	N.D.	N.D.
*ihfA*	1.80 ± 0.04	1.70 ± 0.06	1.97 ± 0.32	2.07 ± 0.22	N.D.	N.D.	N.D.
*ihfB*	1.00 ± 0.00	1.00 ± 0.00	1.00 ± 0.00	1.00 ± 0.00	N.D.	N.D.	N.D.
*mlc*	1.10 ± 0.00	1.70 ± 0.06	2.37 ± 0.49	10.66 ± 2.07	N.D.	N.D.	N.D.
rpoD**	1.00 ± 0.20	0.90 ± 0.20	1.00 ± 0.20	1.18 ± 0.22	0.70 ± 0.20	1.10 ± 0.10	N.D.
*rpoS +*	2.90 ± 0.86	2.20 ± 0.20	2.03 ± 0.07	4.62 ± 0.34	0.10 ± 0.00	0.20 ± 0.00	2.60 ± 0.10

Relative mRNA concentrations of central metabolism and regulatory genes of JM101, PB11, PB12 and their derivatives strains, grown in glucose as the sole carbon source, were determined by RT-qPCR. Most of these values have previously been reported (*) and are presented here for discussion and comparison purposes [9-11]. ArcA/B regulated genes are marked with (+). Data in this table are reported as relative expression levels of the parental JM101 strain, assigning it the value of one (see Materials and Methods). The RT-qPCR values of mutated (**) or deleted (***) genes in the PB12 strain have not been reported, with the exception of *arcB* and *rpoS*. N.D. Not determinate.

### Inactivation of mutated regulatory and putative regulatory genes in the PB11 and PB12 strains. RT-qPCR values of carbon central metabolism genes of the PB11ΔrppH strain

With the aim of understanding the possible roles of some of the regulatory and possible regulatory genes mutated in PB12, as well as in its parental PB11 and JM101 strains (Table 1A), they were individually inactivated by a cassette insertion using the Datsenko-Wanner method [46] (see Materials and Methods), with the exceptions of the *rpoD* gene, since its inactivation is lethal [47], and the *galR* gene, since its regulated target, the *galP* gene, is already overexpressed in the PB11 and PB12 strains [9,23]. Interestingly, as shown in Table 2, individual cassette inactivation of the *arcA*, *rppH*, *yjjU,* and *rssA* genes in the PB11 strain increased their specific growth rates in glucose as the only carbon source, supporting our hypothesis that the inactivation of some of these genes, especially *rppH,* was the result of direct selection during the evolution process for faster growth.

Some of these regulatory genes were also inactivated by the Datsenko-Wanner method in the PB12 strain; the effects on their respective specific growth rate values are shown in Table 4. Knockout inactivation of the *barA*, *arcA*, and *yjjU* genes decreased the μ by about 5%, 10%, and 23%, respectively, while no substantial growth rate difference were observed when the *rna, rssA,* or *ypdA* genes were inactivated.

**Table 4:** Specific growth rates (μ) of the PB12 strain and its derivatives

**Regulatory genes**		
**Strain**	**μ (h**^**-1**^**)**	**%**
PB12	0.44 ± 0.016	100
PB12Δ*arcA*	0.4 ± 0.0038	91
PB12Δ*barA*	0.46 ± 0.001	105
PB12ΔrpoS*	0.38	86
**Possible regulatory genes**		
**Strain**	**μ (h**^**-1**^**)**	**%**
PB12Δ*yjjU*	0.34 ± 0.003	77
PB12Δ*rssA*	0.43 ± 0.018	98
PB12Δ*rna*	0.43 ± 0.035	98
PB12Δ*ypdA*	0.44 ± 0.003	100

*This value has been previously reported [9-11].

Since the inactivation of the *rppH* gene in the PB11Δ*rppH* strain is responsible for a markedly (261%) μ increment, the expression of several genes, including carbon central metabolism, transport and regulators in this strain, as well as in the JM101Δ*rppH* strain, were determined (Table 3). In PB11Δ*rppH*, most of the RT-qPCR values of the central metabolism genes, including TCA (except *pgk*, *fbaA*, *talB*, *pckA*), increased from 2- to 18-fold, as compared to PB11. RT-qPCR values of all genes involved in growth under stress-limited carbon conditions, which most of them are overexpressed in the PB11 strain due to the lack of PTS (such as the *gal* operon, *poxB*, *acs,* and the glyoxylate shunt genes), were also high in the PB11Δ*rppH* strain and, for some genes, the increase was up to 10-fold, as for the *maeB* gene (Table 3) [8-12]. The mRNA levels of all these previously mentioned genes, except for *aceEF*, *fbaA*, *eno*, *pckA*, *pfkA*, *pykA* and *pgk,* in the PB11Δ*rppH* strain were higher than in the parental JM101 strain. Importantly, RT-qPCR values of several of these genes were also increased in the JM101Δ*rppH* as compared to JM101, but in general at lower level, with the exception of the *gapA*, *pgi*, *glk*, *sdhA*, *sdhB*, and *sucAB* genes, which increased more than 3-fold (Table 3). The RT-qPCR values of some of the ArcA/B regulated genes were also highly increased in the PB11Δ*rppH* (Table 3). When comparing the RT-qPCR values of central metabolic genes in the PB11Δ*rppH* strain with those of the PB12 strain, the TCA cycle genes were reduced in the PB12 strain (Table 3). The analysis and possible explanations of these differences is presented in the next section. Remarkably, RT-qPCR values of most of the regulatory genes analyzed were also increased and, in some cases, highly increased in the PB11Δ*rppH* derivative, as compared to the parental PB11, whereas RT-qPCR values of most regulatory genes in the JM101Δ*rppH* derivative were not substantially modified as compared to its parental JM101 strain.

As mentioned, higher RT-qPCR values of most of the genes in the PB11Δ*rppH* derivative are mainly the result of the increase in the mRNA half-life time due to the absence of RppH. However, since higher levels of most of the transcriptional regulators were detected in the PB11Δ*rppH* strain, it is possible that the enhanced expression of some genes could be, in addition of increased mRNA half-life time, the result of higher or lower expression of their regulatory genes.

### Analysis and possible effects of some of the mutated regulatory and putative regulatory genes in the evolved PB12 strain

It has previously been proposed that the point mutation in the *arcB* gene, detected in the PB12 strain, is apparently responsible for diminishing the ArcA/B function as a repressor, since it is known that inactivation of ArcB or enhancing of its ArcA-P dephosphorylating activity, could contribute to the overexpression of ArcA/B regulated genes [9,20-22]. However, in order to explain the particularly higher mRNA levels of most of the TCA cycle and respiratory genes mainly controlled by ArcA/B in the PB11Δ*rppH* strain, as compared to the PB12 strain (except for *mdh* and *nuoN*; Table 3), we now propose a different role for this *arcB* mutation. This mutation is apparently responsible for modifying the ArcA/B repressor function in the PB12 strain by reducing -not enhancing-, its ArcA-P dephosphorylating capacity, which in turn could contribute to higher repression of ArcA/B regulated genes, explaining the reduction in the RT-qPCR values of most of the genes regulated by the ArcA-P in PB12 as compared to the PB11Δ*rppH* strain. Therefore, this change in the *arcB* gene apparently reduced both, transcription of ArcA/B-dependent genes [9,20,21], and metabolic burden, allowing better growth capacities to the PB12 strain as compared to the PB11Δ*rppH* derivative (Table 3). In agreement with this proposition, the knockout inactivation of the *arcA* gene in the PB12Δ*arcA* strain reduced 10% the μ (Table 4), because higher transcription levels of the ArcA/B-controlled genes resulted in this derivative (data not shown) and this was probably sensed as metabolic burden. The same growth diminishing effect occurred in the JM101Δ*rppH* strain, probably due to higher transcription levels of many central metabolism genes, including some of the TCA cycle, which were apparently responsible for reducing 27% the μ (data not shown), as compared to the parental JM101 strain. In agreement with the important role of the ArcA/B regulator, inactivation of *arcA* in the PB11Δ*arcA* strain increase substantially the μ and the transcription levels of most of the ArcA/B-regulated genes as compared to PB11 strain [10] (Table 3). From these results, it is tempting to propose that inactivation of the *arcA* gene in *E. coli* could be used as a tool for allowing better growth capabilities to cells growing aerobically in certain stress conditions, in which the lack of regulation of the TCA cycle and respiratory genes would be an advantage [9,10].

It has been proposed that YjjU could be involved in regulatory processes [32,33]. The inactivation of *yjjU* in the PB11Δ*yjjU* strain increased its μ from 0.13 to 0.16 h^-1^. This 23% increment is not as high as the values obtained with the inactivation of *arcA* (243%) and *rppH* (261%) (Table 2). However, *yjjU* inactivation in the PB12Δ*yjjU* strain reduced its μ 23% (from 0.44 to 0.34 h^-1^), as compared to the parental PB12 strain (Table 4). These results suggest that if this protein really functions as a regulatory factor, as has been proposed, the point mutation could allow stronger capabilities to the cell for faster growth in glucose. Cassette inactivation of *yjjU* is the only case in which a gene knockout increased the μ in the PB11Δ*yjjU* derivative, and reduced the μ in the same percentage in the PB12Δ*yjjU* derivative. This mutation has to be investigated further, initially analyzing the transcription pattern of critical genes in the strain PB12Δ*yjjU* as compared to the parental PB12.

The mutation in the *rpoD* gene is responsible of a conserved valine 482 to isoleucine substitution located in the HTH motif of region 4.2 of RpoD that is involved in the recognition of the −35 promoter region. In the co-crystal structure of region 4.2 of *Thermus aquaticus* with promoter DNA, which is almost identical to the *E. coli*, this position is located at the turn of the HTH motif and does not make any direct contact with the DNA [36]. Thus, it is likely that this particular substitution does not affect the affinity of this sigma subunit for the promoter DNA sequences.

Since the knockout inactivation of the *barA*, *rssA*, *rna* and *ypdA* genes did not modify substantially the μ in the PB11 and PB12 derivatives, it appears that these genes played minor or not role at all in the growth recovery observed in the evolved strain.

## Conclusions

We propose that the deletion event that simultaneously removed the *mutH, rppH*, and part of the *galR* genes, mainly responsible for the faster growth (4x) in glucose, occurred as one of the initial events in the adaptive laboratory evolution process which resulted in the evolved PB12 strain. This deletion caused simultaneously: a) a very high mutagenesis rate due to the removal of *mutH*, in a strain lacking PTS that is already responsible of a carbon stress response, b) higher glucose transport, by increased levels of GalP in this strain lacking PTS, due to the inactivation of *galR*[9,12], and c) higher mRNA levels resulting in enhanced glycolytic and TCA fluxes and better respiratory capacity to the precursor of the PB12 strain due to the absence of RppH.

In addition, lower mRNA levels of most of the ArcA/B regulated genes were detected in the PB12 strain as compared to the PB11Δ*rppH* derivative. This can be explained as an enhanced ArcA-P repressor capacity due to the *arcB* mutation that apparently appeared after the deletion of the *rppH* gene in the evolved strain, allowing lower levels of transcription of ArcA/B-regulated genes.

Knockout inactivation of the *barA*, *rssA*, *rna* and *ypdA* genes in the PB11 and in PB12 strains did not modify substantially the μ of the derivatives, suggesting that each of these mutations alone apparently played minor or no roles at all in the growth recovery in the evolved strain. Some of these changes could in fact be neutral mutations [48].

From these considerations, the evidences indicate that the main reasons for fast growth on glucose are apparently the deletion of the *rppH*, *galR,* and *mutH* genes and, perhaps, the point mutation in the *arcB* gene. These two changes could have been fixed in a short period of time during the fermentation process. Nevertheless, it cannot be ruled out that other point mutation, as those in the *yjjU,* or in the *barA* genes that have not been completely characterized in this study, could also play a minor role in the growth recovery in glucose.

In this study, as in others [4,5], we used two different whole genome sequencing strategies which produced slightly different results. True changes had to be discerned from false positives by conventional Sanger sequencing. Therefore, it is important to emphasize the relevance of using more than one genome resequencing method for this type of studies to have high confidence in the results.

Finally, the results presented here show the physiological plasticity of *E. coli* and could be useful in the design of more robust adaptive laboratory evolution strategies.

## Methods

### Bacterial strains, growth conditions and recombinant DNA techniques

*E. coli* strains JM101 [F’ *traD36 proAB*^*+*^*lac*I^q^*lac*ZΔM15/*supE thi* Δ(*lac-proAB*) *rpoS(33 am)*, PB11 [JM101Δ(*ptsH, ptsI, crr)*:: *kan]* and PB12 (PB11, PTS^-^ Glc^+^) and derivatives have previously been described [7,9-11,38,39]. The derivatives of these strains utilized in this report, in which the *barA*, *yjjU*, *rssA*, *rna*, *ypdA*, and *rppH* genes were knockout inactivated, were obtained by the Datsenko and Wanner method [46], using the oligonucleotides listed in table S3 presented in Additional file 4. All gene disruptions were confirmed by PCR (data not shown). For *inoculums* preparations, strains stored at −72°C in glycerol were inoculated into Luria broth (LB) for overnight growth.

The culture of the PB12 strain that was also utilized for preparing the DNA for genome sequencing, was obtained from the original culture that has been kept frozen in glycerol (Figure 1) [10]. For μ determinations, cells were grown in LB and then inoculated into M9 minimal medium with 2 g/l of glucose as the only carbon source; when the cultures were growing exponentially, they were inoculated into the same prewarmed (50 ml) medium at 37°C and stirred at 300 rpm with a starting optical density at 600 nm (O.D._600nm_)= 0.1. O.D._600nm_ were measured using a Klett/Summerson photocolorimeter, model 800–3. All specific growth rate values presented in Tables 2 and 4 are the averages of at least two independent cultures, each one in duplicate. For RNA isolation and RT-qPCR analyses, duplicate cultures were grown on 1 L fermentors on M9 medium with 2 g/l of glucose as the sole carbon source, at 37°C, stirred at 600 rpm and air flow rate at 1 vvm, with a starting O.D._600nm_ = 0.1. For RT-qPCR determinations cells of the different fermentations were collected in the log phase at O.D._600nm_ = 1 [9].

### DNA extraction from parental and evolved PB12 strains for genomic analysis

Two overnight cultures of the *E. coli* strains JM101, PB11 and PB12 were grown from their frozen original stocks in liquid LB medium. One set of these cultures (not including PB11) of these strains was utilized for DNA purification submitted to RN, and the DNA of the other set was submitted to the UNAM Massive Sequencing Unit, for genome resequencing (see below). DNA was extracted by a maxiprep phenol extraction and ethanol precipitation method [49] and purified with the Pure Link PCR purification kit (Invitrogen, USA). Quality and quantity of extracted DNA was verified as recommended by RN and by UNAM Massive DNA Sequencing Unit.

### Roche NimbleGen Inc. sequencing

DNA samples from the JM101 and PB12 strains were submitted to RN for CGS analysis using *E. coli* K-12 MG1655 (ATC #47076) as the reference strain [40]. The results provided by RN are included in Table 1, Figure 2 and in table S1 presented in Additional file 1.

### Paired and paired end (PE) library construction and GAIIx sequencing

DNA samples from the JM101, PB11 and PB12 strains were submitted to the Massive DNA Sequencing Unit of UNAM for its paired ended (PE) library construction and genome sequencing. PE library was constructed following Illumina Inc. recommendations. Briefly, 5 μg of chromosomal DNA of each strain was fragmented by nitrogen nebulization during 6 min at a pressure of 32 psi. Fragmented DNA was purified using the QIAquick PCR purification kit and resuspended in 30 μl of elution buffer (EB: 10 mM TrisÂ·HCl, pH 8.5). DNA end repairs were performed using a mixture of T4 and Klenow DNA polymerases and T4 polynucleotide kinase for 5’ ends. In order to facilitate the ligation of double stranded adapters, an adenine residue was incorporated at each 3’ end of fragmented DNA before this step using a Klenow exo minus (exo^-^) enzyme and dATP. Illumina Inc. adapters with overhang thymine residues at 3’ ends were ligated at each end of fragmented DNA using 2x rapid ligation buffer (Illumina Inc.) and T4 DNA ligase during 15 min at room temperature. Ligated DNA was purified using a Qiagen MinElute purification kit (Qiagen, USA) and resuspended in 15 μl of EB. Modified DNA pool was loaded on a 2% gel of Ultra Low Range Agarose (Bio Rad Laboratories USA) and ≈500 bp DNA fragments were purified using a QIAquick gel extraction kit. To enrich the adapter-modified DNA fragments, purified DNA was used as template for a 12-cycle PCR reaction (98, 65 and 72°C), using PCR primers PE 1.0 and 2.0 and Phusion DNA polymerase (included in the Illumina Inc. PE sample prep kit). PCR products were purified using a QIAquick PCR purification kit (Qiagen, USA) and eluted in 50 μl of EB. Validation and quantification of the libraries were performed using an Agilent Bioanalyzer 2100 (DNA 1000 chip). Finally, 18 pM of DNA library were used for a PE sequence of 2x36 cycles on a GAIIx instrument that performs sequencing by a synthesis method based on reversible fluorescent terminators accordingly to Illumina, Inc.

### Genome “de novo” assembly and variant identification by Winter Genomics Inc

Low quality reads produced by the Illumina GAIIx method were filtered using the ShortRead 1.8.0 package [50]. Assembly for each strain was performed with the PE-Assembler 1.1 [51]. IMAGE 2.1 [52] was used to close gaps as it locally assembles reads aligning to contig ends. Bowtie 0.12.5 short read aligner [53] was used to align reads to the resulting contigs and unsupported bases were removed with the Biostrings 2.18.0 package. Contigs were re-ordered along the *E. coli* K12 MG1655 genome [40] by using the Mauve 2.3.1. software [54,55]. Then contigs were compared against the reference genome using both Mauve and Murasaki 1.68.6. softwares [56]. Using the PTS operon deletion as marker, it was possible to correctly identify each strain. BLAT v34 software [57] was used to perform alignments of strain PB12, against JM101. VarScan 1.2 software [58] was used to identify variants using the BLAT alignments as input. Ambiguous variants were filtered out using a custom Perl script. For most of the analyses local cluster resources of the Instituto de Biotecnología-UNAM were used. The results provided by WG for JM101 and PB12 strains are included in Table 1 and in table S2 presented in Additional file 2. None of the point mutations detected in PB12 appeared in the genomic sequencing of the PB11 strain (data not shown).

### DNA sequencing of putative mutations by Sanger methodology

DNA regions containing putative mutations in regulatory genes detected by RN and WG were PCR amplified using oligonucleotide primers listed in table S3 presented in Additional file 4, purified by the Pure Link PCR purification kit and sequenced by the Sanger methodology with the Taq FS Dye Terminator Cycle Sequencing Fluorescence-Based Sequencing, in a Perkin Elmer/Applied Biosystems Model 3730. Sequence differences of 14 of the mutations presented in Table 1A were confirmed by examination of the trace data (data not shown).

### RNA Extraction, DNAse treatment of RNA and cDNA synthesis for RT-qPCR analysis

Total RNA from the utilized strains was isolated and purified using the hot-phenol method, with some modifications. Samples containing 50 ml of the different strains growing logarithmically in the fermentor were collected at 1 OD_600nm_. 1 ml of RNA later buffer (Ambion Inc., USA) was added to each sample, mixed and centrifuged 10 min/4°C/5000 rpm. Cells were resuspended with 1 ml of buffer I (0.3 M sucrose, 0.1 M sodium acetate), treated with 20 μl of lysozyme (10 mg/ml in TE buffer) for 10 min at room temperature. 2 ml of buffer II (0.01 M sodium acetate, 2% SDS) were added and the mixtures incubated for 3 min at 65°C. The lysates were extracted with 2 ml of hot phenol and heated for 3 min at 65°C. A second extraction with hot phenol was performed without heating the mixtures. Samples were then extracted with 2 ml of a phenol:chloroform mixture (1:1), precipitated with 0.1 volume of 3 M sodium acetate (pH 5.2) and 2.5 volume of ethanol and centrifuged for 15 min at 4°C/10000 rpm. Samples were then suspended in 300 μl of DNAse and RNase-free water (Ambion Inc, USA) with RNase inhibitor (Fermentas Life Sciences, USA) and extracted twice with 1 volume of chloroform. Finally, samples were precipitated as before and suspended in 300 μl of TE buffer (Ambion Inc, USA). RNA was analyzed on formaldehyde agarose gel for integrity. RNA concentrations were quantified using Nanodrop 2000c (Thermo Scientific); the 260_nm_/280_nm_ and 260_nm_/230_nm_ ratios were examined for protein and solvent contamination. For all samples the 260_nm_/280_nm_ absorbances values were between 1.9-2.0 and in the range of 2.0-2.3 for the 260_nm_/230_nm_ ratio. RNA samples were stored at −70°C. Three RNA extractions and purifications were carried out from three independent fermentations for each strain.

For DNAse treatment, total RNA samples were treated with TURBO DNA-free kit (Ambion Inc, USA) at 37°C for 30 min, following manufacturer’s instructions. To determine whether RNA samples were significantly contaminated with genomic DNA, samples were subjected to conventional PCR with primers for the *arcA* gene (Table S3 presented in Additional file 4). Since these primers were designed to recognize genomic DNA, the presence of a detectable PCR product on an ethidium bromide-stained agarose gel confirmed that the specific RNA sample was contaminated with genomic DNA. Contaminated samples were discarded. PCR reactions were performed with Taq polymerase (Fermentas Life Sciences, USA). The cycling parameters were: 95°C for 5 min, 30 cycles at 95°C for 1 min, 55°C for 1 min and 72°C for 1 min plus an extension step at 72°C for 5 min. Additionally, the DNAse-treated RNA samples were used for RT-qPCR analyses of the same *arcA* gene, using the appropriate oligos *arcA*a (forward) and *arcA*b (reverse)] (Table S3 presented in Additional file 4). As in the PCR case, all utilized samples did not produce a 101 bp amplimer, indicating that small fragments of genomic DNA were not present. cDNA was synthesized using RevertAid^TM^ H minus First Strand cDNA Synthesis kit following the manufacturerÂ´s instructions (Fermentas LifeSciences, USA.). For each reaction approximately 5 μg of RNA and a mixture of 10 pmol/μl of specific DNA reverse primers (b) for the utilized genes, were used. Nucleotide sequences of these genes have been previously published [9-11] or are listed in table S3 presented in Additional file 4. cDNA were used as templates for RT-qPCR assays. cDNAs were synthesized using specific oligonucleotides, since this condition ensures the synthesis of only one copy of cDNA per each RNA molecule [9,59].

### RT-qPCR

RT-qPCR was performed with the ABI Prism 7000 Sequence Detection System and 7300 Real Time PCR System (Perkin Elmer/Applied Biosystems, USA) using the Maxima^R^ SYBR Green/ROX qPCR Master Mix (2X) kit (Fermentas LifeSciences, USA). MicroAmp Optical 96-well reaction plates (Applied Biosystems, USA) and Plate Max ultraclear sealing films (Axygen Inc, USA) were used in these experiments. Amplification conditions were 10 min at 95°C, followed by a two-step cycle at 95°C for 15 sec and 60°C for 60 sec for a total of 40 cycles, to finish with a dissociation protocol (95°C for 15 sec, 60°C for 1 min, 95°C for 15 sec and 60°C for 15 sec). DNA sequences of the primers for specific amplifications were designed using the Primer Express software (Perkin Elmer/Applied Biosystems, USA). Some of these sequences have been previously published [9-11] and the rest are included in table S3 presented in Additional file 4. All RT-qPCR experiments complied with the MIQE guidelines (Minimum Information for Publication of Quantitative Real-Time PCR Experiments) [60,61]. The length of all the utilized oligonucleotides (forward and reverse), was between 18 and 21 nucleotides, with % of GC between 45 to 60 and Tm between 58 to 60°C. The size of all amplimers was 101 bp. The final primer concentration was 0.2 μM, in a total volume of 12 μl. Five ng of target cDNA for each gene were added to the reaction mixture, since higher cDNA concentrations (>10 ng) are not in the dynamic range of the reference *ihfB* gene (see below). Hence the obtained values cannot be correctly normalized for this higher cDNA concentration. All experiments were performed at least in triplicate from three different fermentations, for each gene of each strain, obtaining very similar values (differences <0.3 SD). A non-template control reaction mixture was included for each gene and values appeared for all genes after cycle 31. Standard curves were constructed to evaluate PCR efficiency and all the genes had R^2^ values above 0.9976 with slopes between −3.4 to −3.7. The quantification technique used to analyze data was the 2^-^ΔΔ^Cq^ method described by Livak and Shmittgen [62]. Data were normalized using the *ihfB* gene as an internal control (reference gene). The same reproducible expression level of this gene was detected in all the strains in the conditions in which bacteria were grown and analyzed, since this is the most important characteristic that a reference gene should have in accordance with the MIQE guidelines. Additional file 5 (Figure S2) presents the *ihfB* gene values detected for the utilized strains. These results demonstrate the stability of the expression of this reference gene in all the analyzed derivatives for the used conditions in this report and also on previous reports utilizing these strains and other derivatives [9-11,60].

For each analyzed gene in all strains the transcription level of the corresponding JM101 gene, was considered equal to one, and it was used as control to normalize the data. Therefore, data are reported as relative expression levels, compared to the expression level of the same gene in the JM101 strain. Results presented in Table 3 are the averages of at least three independent measurements of the RT-qPCR expression values for each gene. Values were obtained from different cDNAs generated from two independent bioreactor samples [9].

## Competing interests

The authors have declared that no competing interests exist.

## Authors’ contributions

CA carried out all the molecular, genetic and bacterial growth experiments. CA and RA participated in the fermentor cultures of the strains. CA and NF performed the RT-qPCR analysis. FR-M and AE participated in the genome assembly and variant identification of the strains. CA, AE, EM and FB carried out the data analysis. CA, AE, GG, EM and FB conceived the study and designed the experiments. CA, EM and FB wrote the paper. All authors read and approved the final manuscript.

## Funding

Consejo Nacional de Ciencia y Tecnología (CONACyT/México grants 105782, FONSEC/SSA/ISSSTE/CONACyT 44126, 126793 and INOVAPYMME 137117,155519; Dirección General de Asuntos del Personal Académico-Programa de Apoyo a Proyectos de Investigación e Innovación Tecnológica-Universidad Nacional Autónoma de México (DGAPA-PAPIIT-UNAM) grants IN213508, IN224709, IN205811 and IN221106. The founders had no role in study design, data collection and analyses, decision to publish, or preparation of the manuscript.

## Supplementary Material

Additional file 1Additional file 1 **Table S1.** Mutations in coding regions detected by Roche NimbleGen Inc. This table includes the data provided by Roche NimbleGen Inc. (RN) for the whole genome sequence analysis of the evolved PB12 strain. Section A lists 26 (21+5) non-synonymous point mutations in structural genes that accordingly to RN changed the coding regions of these genes. In fact only in 21 of these genes detected also by Winter Genomics Inc (WG), the mutations occurred (Table 1A). Section B presents the list of the 12 genes included in a large deletion in this strain. This list also includes the genes deleted in the parental PB11 strain, for the construction of this derivative lacking PTS (Figure 2). The table also includes (Section C), the list of the 20 genes in which synonymous point mutations occurred accordingly to RN. Those 16 in common with WG are in bold letters. Click here for file

Additional file 2 **Table S2.** Mutations in coding regions detected by Winter Genomics Inc. This table lists the data provided by Winter Genomics Inc. (WG) obtained for the whole genome sequence of the PB12 strain in comparison to the parental strains JM101 and PB11. Importantly, PB11 strain was sequenced by WG and the same nucleotide sequences as in the parental strain JM101 were determined (data not shown). Therefore all the point mutations detected in PB12 by RN and WG appeared during the laboratory evolution process. Section A includes a list of 27 genes (21+6) in which, accordingly to this company non-synonymous point mutations occurred changing the coding regions in structural genes. 21 of these genes were also detected by RN (Table 1A). The table also includes (Section B) the list of 18 genes in which synonymous mutations also occurred, accordingly to WG. Those 16 in common with RN are in bold letters. Click here for file

Additional file 3 **Figure S1.** Nucleotide sequence of the chromosomal genes fusion that occurred in the evolved PB12 strain. This figure includes the nucleotide sequence of the genomic region where the deletion occurred (Figures 3 and 4) between the ptsP and the galR genes in the PB12 strain. Click here for file

Additional file 4 **Table S3.** Oligonucleotides employed in this study. This table lists the oligonucleotides utilized in this work. Section A shows the oligonucleotides used for DNA sequencing with the Sanger method, including those for the confirmation of the deletion that occurred in the PB12 strain (Figures 2, 3 and 4), the reported mutations provided by RN and WG (Table 1 and Tables S1 and S2 in Additional file 1 and Additional file 2) and the ones employed for gene disruption confirmation. Section B lists the oligos utilized for gene disruption with the Datsenko-Wanner methodology [46]. Section C lists the oligonucleotides utilized for RT-qPCR analysis not previously reported. The sequences of the oligos utilized for the remaining genes listed in Table 3, have been previously published [9-11] (see Materials and Methods). Click here for file

Additional file 5 **Figure S2.** Amplification curves for the *ihfB* gene in different strains. This figure shows the positions of the amplification curves for the *ihfB* gene (Section A) and the Ct values of this gene (Section B), in the different strains employed in this study. As can be seen, all the amplification curves of the *ihfB* gene that has been used as the reference gene for the determination of the RT-qPCR levels, have very similar values. The values presented in the table are from three different fermentations (F1, F2 y F3) for each utilized strain. Since all the values included in section B are very similar, only one third of them are presented (labeled with an asterisk *) in section A. These results demonstrate that the same reproducible expression levels were obtained for the *ihfB* gene in all strains. This is the most important characteristic that a reference gene should have, in agreement to the MIQE guidelines [59-61]. These results corroborate the stability of the expression of the reference *ihfB* gene in these strains in the utilized conditions. (EPS 308 kb) Click here for file
